# The Role of Resolvins: EPA and DHA Derivatives Can Be Useful in the Prevention and Treatment of Ischemic Stroke

**DOI:** 10.3390/ijms21207628

**Published:** 2020-10-15

**Authors:** Nikola Tułowiecka, Dariusz Kotlęga, Piotr Prowans, Małgorzata Szczuko

**Affiliations:** 1 Department of Human Nutrition and Metabolomics, Pomeranian Medical University in Szczecin, 71-460 Szczecin, Poland; ntulowiecka97@gmail.com; 2 Department of Neurology, Pomeranian Medical University in Szczecin, 71-252 Szczecin, Poland; dkotlega@uz.zgora.pl; 3 Department of Applied and Clinical Physiology, Collegium Medicum University of Zielona Gora, 65-417 Zielona Gora, Poland; 4 Clinic of Plastic, Endocrine and General Surgery, Pomeranian Medical University in Szczecin, 72-009 Police, Poland; Pprowans@wp.pl

**Keywords:** resolvin, maresin, DHA, EPA, cardiovascular disease, stroke

## Abstract

Introduction: Most ischemic strokes develop as a result of atherosclerosis, in which inflammation plays a key role. The synthesis cascade of proinflammatory mediators participates in the process induced in the vascular endothelium and platelets. Resolvins are anti-inflammatory mediators originating from eicosapentaenoic acid (EPA) and docosahexaenoic acid (DHA), which may improve the prognosis related to atherosclerosis by inhibiting the production of proinflammatory cytokines, limiting neutrophil migration, or positively influencing phagocytosis. Although clinical trials with resolvin in humans after stroke have not been realized, they may soon find application. Aim: The aim of the study was to review the available literature on the scope of the possibilities of the prevention and treatment of stroke with the use of resolvins, EPA and DHA derivatives. Materials and methods: The review features articles published until 31 January 2020. The search for adequate literature was conducted using the keywords: stroke and resolvins. Over 150 articles were found. Studies not written in English, letters to the editor, conference abstracts, and duplicate information were excluded. Results: In several studies using the animal model, the supplementation of resolvin D2 decreased brain damage caused by myocardial infarction, and it reversed the neurological dysfunction of the brain. A decrease in the concentration of proinflammatory cytokines, such as TNF-α, Il-6, and Il-1β, was also observed, as well as a decrease in the scope of brain damage. In the context of stroke in animals, the treatment with resolvin D2 (RvD2) (injection) has a better effect than supplementation with DHA. Conclusions: Resolvins are characterised by strong anti-inflammatory properties. Resolvins improve prognosis and decrease the risk of developing cardiovascular disease, consequently lowering the risk of stroke, and may find application in the treatment of stroke.

## 1. Introduction

Ischemic stroke is currently the most frequent cause of disability in adults and one of the most common causes of death in the USA [1]. Despite the fact that the mortality rate resulting from strokes has decreased in the recent decade in the USA, it still constitutes a large percentage of the population [1]. In the case of European countries at the beginning of the 21st Century, the rate of cases was between 95 and 290 per 100,000 people a year (the data are from 2000–2010 and were extracted from the Register of Strokes or Stroke Reports), so it is in the range of 0.29%. A higher prevalence of this illness was observed in Eastern Europe, less in the south [2]. The geographical differences associated with the prevalence of strokes may be related to environmental conditions, diet type, genetic factors, lower income, and thus, limited access to and the lower quality of the healthcare system [2]. The prevalence increases with age, especially after the age of 80, and the stroke incidence is more frequent in men than women over 60 years of age, as is also confirmed by European studies [1,2]. Fatigue, cognitive impairment, and lower quality of daily life can be present even after a minor stroke [3]. Continued and complex posthospitalization care, including treatment for depression and increased social support, covers the vast financial resources of healthcare [4]. Moreover, the perceived impact of stroke becomes more prominent with time, even for persons with mild-to-moderate stroke [5]. There are two main types of stroke leading to focal neurological deficit: ischemic and haemorrhagic stroke, which constitute 80–85% and 10–15% of all strokes, respectively. The standard treatment of ischemic stroke is to administer thrombolytic drugs to the patient or mechanical thrombectomy, while the haemorrhagic stroke methods are limited [6,7]. Current studies show that the level of DHA derivatives significantly decreases in the early post-stroke stage (up to seven days) compared to the control group. It is also related to the decrease of its precursor, DHA. Moreover, EPA was also lowered, but not resolvin E1 (RvE1) [8]. The same authors found that there was a relationship between the severity of depressive symptoms in stroke patients and the level of eicosanoids and free fatty acid (measured seven days after the incident and six months later). Patients with lower levels of DHA and its derivative (RvD1) had worse results by the Beck Depression Inventory-II [9]. The aim of this study is to overview the risk factors associated with stroke, the role of inflammation in stroke and the effects of EPA and DHA derivatives on the aspects of stroke pathomechanisms. We also discuss the potential beneficial effects of resolvins in the prevention and treatment of stroke taking into consideration the pre-stroke period and the acute phase of stroke. As clinical trials have not been conducted in humans, the last of the goals will be the discussion of the examples of experimental treatment in the animal models.

## 2. The Risk Factors for Developing Stroke

The risk factors of stroke development are associated both with some diseases, such as hypertension, dyslipidemia, obesity, and diabetes, as well as lifestyle (not enough physical activity, bad diet, smoking tobacco, and the overuse of alcohol) [10]. The modifiable factors listed above are responsible for as much as 90% of the possibility of developing stroke, whereas the remaining 10% are non-modifiable factors [11]. The latter include age, male gender, race and ethnicity, positive family history, socioeconomic status, and genetic factors (e.g., gene mutations of coagulation factors, proteins involved in lipid or homocysteine metabolism) [12].

One of the most important interventions that can prevent ischemic stroke is the treatment of hypertension. Hypertension is defined as systolic blood pressure ≥ 140 mm Hg or diastolic blood pressure ≥ 90 mm Hg. The occurrence of hypertension in stroke patients is more than 70% [13]. The effectiveness of treatment in the prevention of this illness has been confirmed more than once in randomised clinical studies: the risk of another stroke decreased by about 30% [13]. Studies also show a positive influence of the treatment of lipid disorders in patients after ischemic stroke, particularly when accompanied by an excessive level of LDL-C and an insufficient level of HDL in the blood serum of these patients. Treatment with statins decreased the risk of a subsequent ischemic stroke by 3.5–18% [11,14]. In patients after stroke, there are often problems with glucose metabolism disruptions: type 2 diabetes or impaired fasting glycemia (glucose levels: 100–125 mg/dL) were observed in about 70% of patients with ischemic stroke [13]. In persons suffering from diabetes, there is a risk of not only subsequent strokes, but also of the first ischemic stroke [15]. Obesity is another frequent disorder in stroke patients (44% of patients). In obese patients, most frequently, there are several connected risk factors related to stroke, including the abovementioned type 2 diabetes, hypertension, and dyslipidemia. Numerous studies indicate that obesity is associated with increased mortality, especially in young, post-stroke patients [16]. On the basis of the BMI analysis of stroke patients, it has been established that obese and overweight patients (BMI ≥ 25 kg/m^2^) are characterised by much lower mortality in comparison to patients with correct BMI (<25 kg/m^2^). This is why it is suggested that BMI (the interpretation of <25/ kg/m^2^ considered as correct) is not an accurate indicator of the presence of obesity, particularly in elderly people. The study also did not take into account the distribution of adipose tissue, which is also an important factor determining the mortality in stroke [17,18,19,20]. In summary, modifiable risk factors of stroke pose the greatest risk of stroke and complications associated with this disease. The modifiable risk factors of stroke affect the inflammatory system leading to multidirectional, inflammatory changes and the development of atherosclerosis [21].

## 3. Inflammation in Stroke

Inflammation constitutes the significant pathogenetical factor of ischemic stroke because it is associated with the development of atherosclerosis. During a stroke, the death of neurons progresses very rapidly, from a few minutes to up to a few hours. This is caused by energy deficit, the lack of ion balance, the ineffectiveness of mitochondria, and the activation of intracellular lipases, proteases, and ribonucleases, which cause a rapid breakdown of the structural elements of cells and their integrity [21,22]. Immunological response begins locally in the vessel and, in the case of ischemic stroke, in ischemic brain cells. The inflammation cascade is activated immediately after the obstruction of blood vessels. Vascular stasis causes stress in the vascular endothelium and activates platelets. The distribution of P-selectin takes place on the cell surface, which is key for the deceleration of circulating leukocytes [21]. P-selectin binds with leukocytes, further increasing ischemic damage. Other adhesive molecules, such as E-selectin, intercellular adhesion molecule-1, and the vascular cell adhesion molecule-1, play a key role in the coordination, recruitment, adhesion, and migration of leukocytes in blood [23]. The basis of inflammation in ischemic stroke is the adhesion of neutrophils to endothelium cells and the cascades of blood coagulation, additionally stimulating inflammation signals. Thrombin induces the expression of adhesive molecules on endothelial cells and activates the C3 and C5 constituents of the complement system, and it may disrupt the function of the endothelial barrier. It forms a strong anticoagulation and anti-inflammatory complex, and it leads to the activation of monocytes and the complement system [21,22,23,24]. Inflammation mediators are spread throughout the entire body, leading to a systemic inflammatory response and, later, immunosuppression aiming at the suppression of a potentially damaging proinflammatory environment [24]. In experimental stroke, the systemic inflammatory response signal is characterised by an increased level of cytokines in the serum (interleukin-6, interferon-γ, CXC-chemokine ligand) and the increased production of inflammatory mediators in immune cells (TNF, IL-6, IL-2, CXC-chemokine ligand 12) within a few hours of ischemia [22,23,24]. Most of these parameters return to initial output levels within 24 h after stroke. The level of IL-6 in the serum is positively correlated with the exacerbation of stroke [25]. Ischemic stroke is associated with the presence of a strong inflammatory response in which arachidonic acid (AA) also plays a key role. Derivatives are synthesised by using three pathways, 5LOX, 15LOX, and COX1, 2 together with prostaglandins. It seems that 12LOX plays only a minor, insignificant role in this process [26,27,28].

Omega-3 PUFAs represent the precursors of lipid mediators, including resolvins, maresins, and protectins, which are collectively termed “specialized pro-resolving mediators” (SPMs) [29]. EPA and DHA can be incorporated into platelet phospholipid membranes at the expense of AA, and thus can decrease the synthesis of AA-derived metabolites and reduce the platelet aggregation [30]. The paths leading to the synthesis of EPA derivatives, DHA in particular, are also amplified as a result of the increased use of resolvins. There is growing evidence pointing to different mechanisms in the pathogenesis of stroke, but its progression depends on the intensity of the inflammation. Therefore, resolvins may improve the prognosis in numerous illnesses, including atherosclerosis, which is the main cause of stroke. The atherosclerotic plaque is characterised by a high presence of oxidised lipids and low-density lipoproteins, as well as the accumulation of monocytes and neutrophils [31]. Due to the dominating role of the inflammatory process in the pathogenesis of ischemic stroke, we concentrate in our work on the analysis of the role of resolvins in this type of stroke.

## 4. Resolvins: Synthesis

Excessive or uncontrolled inflammatory processes in the body contribute to the development of many illnesses that influence numerous cells and mediators. In recent years, there have been intensive studies regarding the identification of proinflammatory mediators originating from the enzymatic oxidation of polyunsaturated omega-3 acids: eicosapentaenoic acid (EPA) and docosahexaenoic acid (DHA) [32]. n-3 α-linolenic acid (ALA) is an important constituent of the cell membrane and is transformed into EPA with an efficiency of about 8%, whereas DHA is transformed with an efficiency of about 0.5%. The human ability to enzymatically convert ALA into DHA or EPA is limited, so they should be supplemented with diet [33]. The main source of DHA in diet are sea fish and seafood, out of which wild fish (sea fish) contain more omega-3 acids than farmed fish due to the fact that most of them feed on phytoplankton and zooplankton, which are rich in these fatty acids [33,34]. Most plant seeds and oils, e.g., rapeseed, soy, corn, and sunflower, are mainly a source of omega-6 acids, with a small amount of omega-3. The exceptions are the seeds of chia, flax, and hemp, which are rich in omega-3 acids, and they should be used for diet supplementation [33,35]. Studies show that EPA and DHA can have a positive influence on the resolution of atherosclerotic inflammation by reducing the synthesis of proinflammatory lipid mediators [30]. One such mediator is resolvins, which are synthesized with the involvement of cyclooxygenase pathways (COX) and lipoxygenase pathways (LOX) [32]. EPA serves as a substrate for the creation of resolvins E, whereas resolvins D are formed from DHA [36].

**Figure 1:**
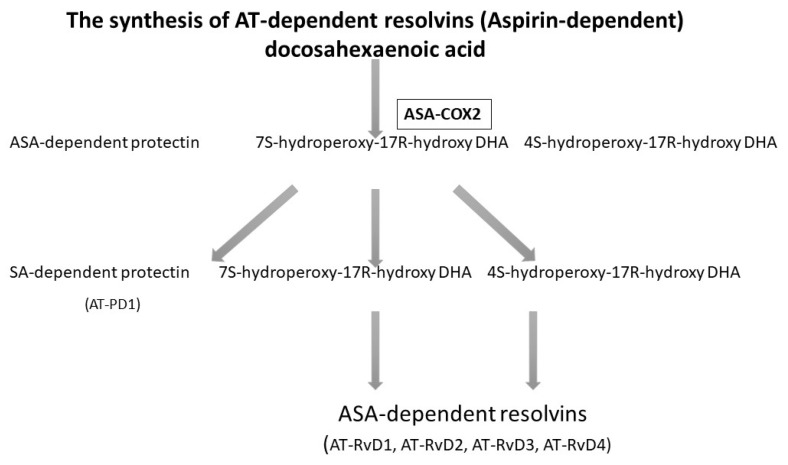
The biosynthesis of resolvins D with the presence of acetylsalicylic acid (ASA) [38,39]. Rv, resolvin.

Acetylsalicylic acid (ASA), known as aspirin, acetylates cyclooxygenase-2 (COX-2) and enables the biosynthesis of precursors for anti-inflammatory mediators. Experimental studies show that, when influenced by ASA, EPA in mice is transformed into completely new products with anti-inflammatory properties. Because DHA has a cardioprotective effect, is abundantly present in the brain and the retina, and has an influence on numerous physiological processes, analyses were conducted to determine whether during inflammation, DHA was used in the treatment of ASA [37]. The analysis revealed that in mice treated with ASA and DHA, new bioactive docosanoids were formed, such as 17R-hydro(peroxy)-docosahexaenoic acid (17R-H (p) DHA), which is then transformed into resolvins D1-D4 thanks to COX-2 [37], as presented in Figure 1.

**Figure 2:**
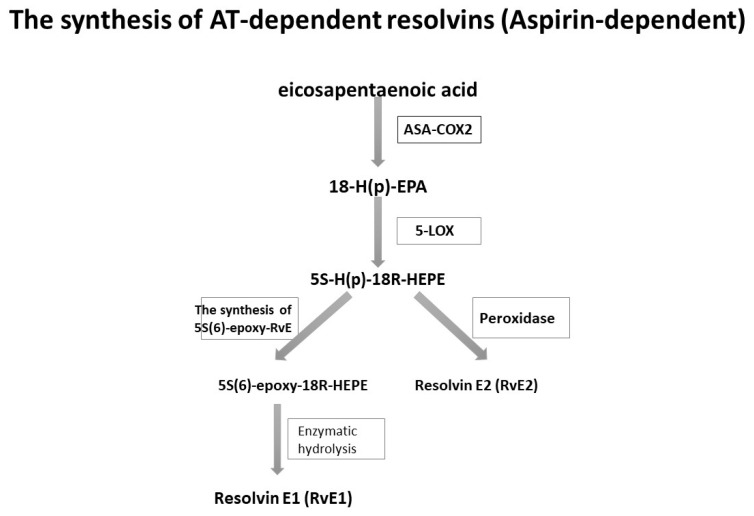
The biosynthesis of resolvins E with the participation of ASA [38,39].

A similar observation was made in the case of the synthesis of resolvins E. In mice treated with ASA, EPA was transformed into 18R-hydro (peroxy)-eicosapentaenoic acid (18R-H (p) EPA) thanks to the capabilities of the ASA-COX-2 enzyme [39,40]. Subsequently, this acid, in conjunction with 5-LOX, underwent further transformations into resolvins E, as presented in Figure 2.

It has been proven that resolvin E1 (RvE1) significantly decreases inflammation and the migration of neutrophils, activating the resolution of inflammation [38]. Moreover, it decreases the release of pro-inflammatory cytokines, mainly interleukins (Il-1, Il-6, Il-12, Il-17, and Il-23). One of the most studied cytokines in the cerebrospinal fluid and blood of stroke patients is the proinflammatory IL-6 [38,39,40]. However, there is still not many data on the mechanism of pro-inflammatory cytokines’ activities in the early stage of stroke (4–6 h after the induction of experimental stroke). It is known that TNF-α, Il-1, and Il-6 increase in amount, even 40–60 times, within 24 h after a stroke [40]. Apart from the decrease in the release of pro-inflammatory cytokines, resolvin E1 also inhibits inflammatory angiogenesis and stimulates phagocytosis through macrophages. The strong anti-inflammatory activity of this resolvin was also observed in an in vivo study that focused, among others, on the kidney damage caused by ischemia, the decrease of inflammation in white adipose tissue (in mice), the reduction of fatty liver, and the improvement of insulin sensitivity. Studies show a strong protective effect of resolvins E not only in the circulatory system, but also in the respiratory system, where they facilitate apoptosis, promoting the resolution of respiratory tract inflammation caused by contact with an allergen [41]. It is also known that resolvin E2 (RvE2) is characterised by weaker anti-inflammatory activity in comparison to RvE1, but it still has a positive impact on phagocytosis, the regulation of the functioning of integrins located on the leukocyte surface, facilitating their migration to the site of inflammation, and also limiting the recruitment of granulocytes. Moreover, it has an influence on the production of the interleukin-10 (Il-10) anti-inflammatory cytokine by macrophages [42]. Il-10 is an anti-inflammatory cytokine that inhibits the expression of pro-inflammatory cytokines and regulates the innate immune response. In studies conducted on rats, Il-10 decreased the scope of ischemic stroke and the damage caused by cerebral artery obstruction [43]. It has also been demonstrated that lower levels of Il-10 are associated with an increased risk of stroke [44].

Resolvins D are created from DHA, and similarly to resolvins E, they have a strong anti-inflammatory effect. They contribute to the decrease in the migration of neutrophils. Furthermore, the activity of resolvin D1 (RvD1) is a mechanism that limits the damage that free radicals cause to tissues during oxygen explosion while the microorganism removal takes place. Resolvin D1 also inhibits the production of the Il-1β pro-inflammatory cytokine [44]. This is an acute-phase pro-inflammatory cytokine that serves as a chemoattractant for neutrophils, natural killer cells (NK), and T-lymphocytes. Most probably, it plays a key role already after the occurrence of stroke, and it may be a marker for the long-term changes after a stroke or brain damage [45]. Studies show that resolvin D4 promotes the synthesis of other D-series resolvins involved in the inhibition of inflammation. Moreover, it decreases the infiltration of neutrophilic granulocytes, and it increases the number of monocytes in the case of blood vessel thrombosis [46].

## 5. Receptors for Resolvins

So far, four G-protein coupled transmembrane receptors have been identified serving as receptors for resolvins D and E: DRV1/GPR32 (resolvin D1 receptor/G-23 protein coupled receptor), DRV2/GPR18 (resolvin D2 receptor/G-18 protein coupled receptor) for D-series resolvins, ERV1/ChemR23 (resolvin E1 receptor/chemerin receptor 23) and the leukotriene B_4_ receptor 1 (BLT1) for E-series resolvins (resolvin E1 and resolvin E2) [47,48]. The latest studies conducted on mice revealed the significant therapeutic role of these receptors in the course of atherosclerosis via the ability to induce the resolution of inflammation in cardiovascular diseases [47]. Resolvins D1 and D3 transmit signals through the DRV1/GPR32 receptor, which is also activated by resolvin D5 and the analogues of D-series resolvins released through aspirin. The receptor undergoes expression in macrophages, increasing phagocytosis. D-series resolvins, through the DRV1/GPR32 receptor, also regulate the functioning of the immune system, preventing the differentiation of T-lymphocytes towards Th1 and Th12, and promoting regulatory cell (Tr) formation [47,48]. It has been proven that the atherosclerotic process features the transformation of vascular smooth muscle cells (SMCs) into a proliferative and migratory phenotype [49]. As a result, SMCs migrate to the site that was altered by atherosclerosis and stabilise the atherosclerotic plaque, forming its main constituent. Therefore, D-series resolvins may have a positive anti-inflammatory influence on blood vessel walls due to the presence of the DRV1/GRP32 receptor also in the vascular endothelium and SMCs [50,51]. Moreover, RvD3 is also associated with the DRV1/GPR32 receptor, which promotes macrophage phagocytosis [52]. Currently, there are no in vivo studies oriented towards the impact of the DRV1/GPR32 receptor on cardiovascular diseases due to the lack of a similar receptor in the studied mice. However, it seems that it can play a significant role in humans [53].

The DRV2/GPR18 receptor was detected in immune cells with various functions. It participates in the development of CD8a lymphocytes in the small intestine. In mice with a decreased amount of this receptor, we observed a smaller number and the migration ability of immune cells [54]. The development of CD8 lymphocytes is significant in the immune therapy of neoplasms, as well as in the treatment of inflammatory bowel diseases and viral infections. The DRV2/GPR18 receptor is also present in skeletal muscles. It has an influence on the resolution of inflammation: studies indicate that resolvin D2, after combining with the DRV2/GPR18 receptor, participates in the recruitment of granulocytes [54]. It has also been proven that DRV2/GPR18 undergoes expression in the heart of mice, particularly in cardiomyocytes. The chronic activation of this receptor leads to a decrease in blood pressure and an improvement of the functioning of the left ventricle in mice [51]. A combination of resolvin E1 and the ERV1/ChemR23 receptor stimulates phagocytosis, contributing to the reduction of inflammation and atherosclerotic processes in the arteries. Studies indicate that RvE1 may indirectly influence the development of cardiovascular diseases also through the change in metabolic factors because increased expression of the ERV1/ChemR23 receptor in fat tissue and a simultaneous decrease in the level of proinflammatory cytokines were observed [51,52]. The BLT1 receptor is expressed on human neutrophils, eosinophils, monocytes, macrophages, mast cells, dendritic cells, and T cells [48]. Both resolvins (RvE1, RvE2) are antagonists of the BLT1 receptor and have counterregulatory effects that lead to the inhibition of neutrophil chemotaxis, calcium mobilization, and NF-kB activation [55,56]. The types of resolvins and their roles are presented in Table 1.

## 6. The Role of Resolvins in Stroke

Though preliminary studies indicate a decrease in the risk of cardiovascular diseases thanks to the introduction of omega-3 acid supplementation, large double-blind studies did not show clear beneficial effects [26]. Omega-3 fatty acids may contribute to the reduction of inflammation both through the reduction of pro-inflammatory factors, as well as through the stimulation of the resolution of inflammation. The potentially protective effect of these acids in cardiovascular diseases, including atherosclerosis and stroke, may refer to their influence on lipid metabolism, thrombosis, and the abovementioned inflammations, which are the risk factors of stroke [26,46].

Dong et al. conducted studies on experimental models: mice with ischemic stroke caused by the obstruction of the middle cerebral artery [57]. RvD2 was supplied to the brain of mice in the form of nanoparticles. The results showed that RvD2 nanoparticles aim precisely at the inflammation of endothelium in the brain. After the supply of RvD2, there was a decrease in the concentration of pro-inflammatory cytokines, such as TNF-α, IL-6, and IL-1β. Furthermore, in mice treated with RvD2, the volume of brain damage after a stroke decreased to 16%, in comparison to 46% in the control group. After the intraperitoneal injection of this resolvin, a decrease of inflammation, a reversal of the formed brain damage, and a reversal of the neurological dysfunction of the brain were observed [57]. The available data points to the benefits of using resolvins in ischemic stroke. The supply of RvD2 also increased the amount of GPR18 protein, which is a receptor for RvD2, particularly in neurons and brain endothelial cells [57,58]. In the animal model of ischemic stroke in rats, the effective dose of RvD2 was 50 µg/kg or 100µg/kg. The beneficial effects were elucidated by the decrease in the volume of the infarcted area of the brain, as well as by the anti-inflammatory properties. The exogenous RvD2 reduces the release of TNF-α and IL6 in the brain, decreases the infarct area, and protects the neurons and endothelial cells of the blood-brain barrier (BBB) from apoptosis and necrosis, maintaining the BBB’s integrity [54,59].

Correia et al. proved that preliminary DHA treatment may successfully improve the process of memory recovery in rats with ischemic stroke. In the context of stroke, treatment with RvD2 (administered directly in the form of an injection) had better treatment results [55,60]. The basis for inflammation in ischemic stroke is the adhesion of neutrophils to endothelium cells. Studies show that RvD2 can decrease the interaction between leukocytes: endothelium cells, as well as the production of cytokines. This happens through the induction of the creation of nitrogen oxide in endothelium cells in order to decrease this interaction [57]. Similar results pointing to the reduction of the concentration of pro-inflammatory cytokines by resolvins were achieved by Xu et al. [61]. They determined that RvD1 prevents the lipopolysaccharide-induced (LPS induced) inflammatory response in microglia cells of mice (in vitro). The LPS-activated microglia cells cause a release of pro-inflammatory factors through the interaction of LPS-TLR4 (lipopolysaccharide-Toll-like receptor-4). After the supply of RvD1, the expression of TNF-α and IL-1β was stopped, and the process of forming pro-inflammatory factors in microglia cells did not occur in the studied mice [61]. According to the study of related signaling pathways, RvD1 attenuated LPS-induced microglia NF-κB activation and MAPK phosphorylation and inhibited the transcriptional activity of protein-1 activator [60,61].

In other studies conducted on mice, it was demonstrated that the supply of RvD2 supported the activity of macrophages, contributing to the increase in the stabilisation of atherosclerotic plaque. The treatment with RvD2 and maresin R1 decelerated the progression of atherosclerosis without influencing body mass, the level of lipids in the plasma, and the number of blood cells of the studied mice [62].

Macrophages in blood have the ability to adapt to the changes in their surroundings due to the fact that they react to receptors and signalling particles. It is usually not possible to clearly determine their phenotype because they swiftly adapt to changes in their surroundings. Macrophages with the M1 phenotype dominate in the early inflammation response: pro-inflammatory pathways are activated in cells. On the other hand, macrophages with the M2 phenotype participate in regeneration processes after inflammation, including in atherosclerotic processes [63,64]. Studies confirmed that in mice supplied with RvD2 and MaR1, the phenotype of macrophages changes from pro-inflammatory M1 into M2, the latter playing an important role in maintaining homeostasis against inflammation and atherosclerosis, as well as supporting regenerative processes [65,66,67].

Additionally, in a study conducted on a mice model of atherosclerosis, it was determined that the supply of resolvin E1 (RvE1) significantly decreases atherosclerotic changes by inhibiting the expression of TNF-α, without changing the amount of macrophages. When supplying RvE1 orally once per day for 16 weeks, there was a decrease in atherosclerotic changes in mice by 35%, and they were classified as mild. Moreover, RvE1 did not have an influence on the level of cholesterol in the plasma [67]. The potential effects of resolvin injections can be expected even faster, because after the intraperitoneal administration of RvD1, its plasma level peaks at one hour, stays constant at three hours, and returns to baseline after 36 h. Such an observation is promising for potential clinical use in humans, as it could be used as an option for treatment in the acute phase of stroke [68].

**Figure 3:**
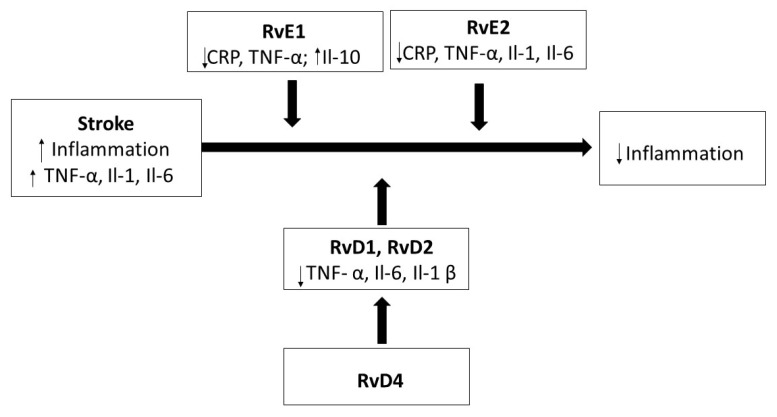
The effect of resolvins on inflammation after a stroke.

Similar results were achieved in rabbits with a high-cholesterol diet, in which the supply of RvE1 caused a decrease in atherosclerotic changes in the aorta and a significant decrease in C-reactive protein (CRP), which, as an acute-phase protein, appears in blood during an inflammation, becoming an inflammation marker [69]. The aggregation of platelets and thrombosis are two factors that, similarly to atherosclerosis and inflammation, support the development of stroke. However, in recent studies, it has been demonstrated that resolvins may also prevent the aggregation of platelets and induce the extension of blood vessels [70]. The hypothetical effects of resolvin after stroke are shown in Figure 3.

Although no studies on the effects of resolvins have been found in humans after stroke, a great deal of research has been done on the effects of omega-3 fatty acids. Yokoyama M. et al. conducted studies on the effect of omega-3 fatty acid supplementation on the prevention of cardiovascular diseases [71]. This randomized placebo-controlled trials proved that EPA supplementation (1.8 g/24 h) may reduce the risk of coronary events by 19% [71]. Similar results were obtained by Bhatt D. et al. in a randomized, placebo-controlled trial (REDUCE-IT) [72]. They studied the effects of EPA supplementation (4 g of icosapent ethyl-EPA ethyl ester/24h) on cardiovascular risk in patients with cardiovascular risk factors. The incidence of cardiovascular incidents and deaths from cardiovascular causes were significantly lower in the group that was supplemented with EPA [72]. The opposite results were presented in the ASCEND and Risk and Prevention trials. The supplementation with EPA and DHA or omega-3 fatty acids in the amount of 1 g per day does not significantly reduce the overall and cardiovascular morbidity of patients with cardiovascular risk factors [73,74].

Clinical studies suggest that omega-3 fatty acids may play a beneficial role in preventing CVD episodes when administered at higher doses. However, more research is needed on resolvins, especially in regard to primary and secondary prevention, as well as in the treatment of the acute phase of stroke in humans.

## 7. Conclusions

Excessive or uncontrolled inflammatory processes contribute to the development of many illnesses. The significant role of omega-3 acids, out of which resolvins with anti-inflammatory influence are formed, was proven in the treatment of cardiovascular diseases, including stroke. It has been confirmed that resolvins have a positive influence on the resolution of inflammation by synthesizing the anti-inflammatory mediators and inhibiting the synthesis of pro-inflammatory mediators. As a result, resolvins influence the improvement of prognosis in stroke cases, but the studies were mostly carried out on rodents.

The limitation of the use of resolvins in stroke patients is mainly connected with the safety profile; thus, there should be a standard safety protocol of the clinical studies performed. The initial, promising treatment with the use of resolvins in animal models may be limited by the potential disadvantages of such a treatment in humans. Secondary intracerebral haemorrhage is a serious, harmful complication of ischemic stroke itself or the thrombolytic therapy in such patients, especially when, additionally, antiplatelet or anticoagulant treatment is used. Taking into consideration the interaction with the inflammatory system, platelets, adhesion molecules, and BBB stability, the risk of haemorrhagic complications after resolvins’ administration should be cautiously analysed.

The future direction for resolvin therapy in humans should start with the analysis of the safety, then the bioavailability and effectiveness need to be established. Rapid bioavailability may give a chance that the beneficial effects of resolvins’ administration would be observed in stroke patients directly after the onset. If the safety profile is granted, we suggest testing such treatment in the time-window as fast as possible after the ischemic stroke onset. This seems to be important, because secondary to brain tissue ischemia, the inflammatory, cytotoxic cascade appears and could potentially be inhibited at an early stage by the use of resolvins. The protective effect on the BBB is also of note, as the cytotoxic cascade after stroke disrupts this barrier. Different neuroprotective strategies have been discussed and tested, but this type of treatment is a promising method of neuroprotection and treatment. The prolonged effect of resolvins would also be beneficial by means of reducing the risk of recurrent stroke or malignant brain oedema, which are serious complications of ischemic stroke. We also suggest taking into consideration the interaction of resolvins with other drugs that can affect the inflammatory system. A number of stroke patients are treated with the use of statins or hypotensives that can interact with resolvins within inflammation.

The mechanism of resolvins’ activity takes place through phagocytosis, the decrease in the migration of neutrophils, the reduction in the synthesis of pro-inflammatory cytokines, and the strengthening of the resolution of the inflammatory process. However, further studies are necessary to determine the existing mechanisms in humans and to determine the efficiency, pharmacokinetics, and safety profile of the preventive or therapeutical dose of resolvins and the way of administration.

## 8. Method of Article Search

The review takes into account articles published until 31 January 2020. The literature was searched for in PubMed and Embase databases using the following keywords: resolvin, stroke, and cardiovascular disease. We analysed 153 articles, and 56 of the works were included in the study. Studies not written in English, letters to the editor, conference abstracts, and duplicate information were excluded. Another 18 studies were included by analysing the individual sections of this review. All included studies were screened and discussed by the authors until a general consensus was reached.

## Figures and Tables

**Table 1:** Characterization of resolvin subtypes and their receptors.

Omega-3	Resolvin Subtypes	Corresponding Receptors	Localisation	Function
EPA	RvE1	ChemR23 (ERV, CMKLR1)	Chemerin receptor 23 is expressed on NK cells, ILCs, macrophages, dendritic cells, and epithelial cells	stimulation of phagocytosis decrease in the level of proinflammatory cytokines
		BLT1		
	RvE2	BLT1	Leukotriene LTB4 is expressed on human neutrophils, eosinophils, monocytes, macrophages, mast cells, dendritic cells, and T cells	reduction in neutrophil mobilization
DHA-	RvD1	ALX/FPR2	Expression on neutrophils, macrophages, monocytes, macrophages, and T cells	increase of phagocytosis prevention of the differentiation of T-lymphocytes towards Th1 and Th12, promotion of regulatory cell (Tr) formation
		DRV1/GPR32	The G-23 protein coupled receptor is expressed on human neutrophils, lymphocytes, macrophages, and monocytes, as well as vascular tissues	
	RvD2	DRV1/GPR32		development of CD8a lymphocytes in the small intestine migration ability of immune cells recruitment of granulocytes decrease in blood pressure
		DRV2/GPR18	The G-18 protein coupled receptor is expressed on human and murine neutrophils, monocytes, and macrophages	
	RvD3	DRV1/GPR32	The G-23 protein coupled receptor is expressed on human neutrophils, lymphocytes, macrophages, and monocytes, as well as vascular tissues	promotion of macrophage phagocytosis
	RvD4	G protein-coupled receptors: no data		inhibition of metastases and induced T cell responses
	RvD5	DRV1/GPR32	The G-23 protein coupled receptor is expressed on human neutrophils, lymphocytes, macrophages, and monocytes, as well as vascular tissues	expression of macrophages increase of phagocytosis.
